# Association between habitual physical activity (HPA) and sleep quality in patients with cystic fibrosis

**DOI:** 10.1007/s11325-020-02130-0

**Published:** 2020-07-14

**Authors:** Sarah Dietz-Terjung, Wolfgang Gruber, Sivagurunathan Sutharsan, Christian Taube, Margarete Olivier, Uwe Mellies, Cordula Koerner-Rettberg, Stefanie Dillenhöfer, Florian Stehling, Matthias Welsner

**Affiliations:** 1 Department of Pulmonary Medicine, University Hospital Essen - Ruhrlandklinik, Adult Cystic Fibrosis Center, University of Duisburg-Essen, Tueschener Weg 40, 45239 Essen, Germany; 2 Pediatric Pulmonology and Sleep Medicine, Cystic Fibrosis Center, Children’s Hospital, University of Duisburg-Essen, Essen, Germany; 3 Department of Pediatric Pneumology, University Children’s Hospital, Ruhr University Bochum, Bochum, Germany

**Keywords:** Cystic fibrosis, Adults, Adolescents, Children, Actigraphy, Sleep quality, Habitual physical activity

## Abstract

**Purpose:**

Sleep disturbances and poor sleep quality are known to be present in patients with CF. Regular physical activity plays an important role in the treatment of CF patients due to its positive influence on progression of disease and quality of life. The aim of this work is to create a home-based sleep and activity profile and to investigate the influence of habitual physical activity (HPA) on sleep quality in children, adolescents, and adults with CF.

**Methods:**

A total of 109 CF patients (64 male, mean age 22.7 ± 12.0 years; mean ppFEV1 63.0 ± 26.7) were equipped with an actigraph for a home-based collection of data on sleep and activity over 4 weeks.

**Results:**

Age, FEV1, and BMI affect sleep and activity in CF patients. Especially younger age and higher FEV1 show a great influence on certain aspects of sleep (SE, TST, TIB, WASO, # of awakenings) and activity and its different intensities. General HPA does not affect sleep, but there is a strong correlation between times spent in vigorous to very vigorous intensities and better sleep quality.

**Conclusion:**

Besides younger age and higher FEV1, daily activity in higher intensities influences sleeping behavior of CF patients in a positive way. Patients with poor sleep quality and sleep disturbances possibly benefit from an intensification of physical activity in the home environment.

**Trail registration:**

number: 14–6117-BO (University Duisburg-Essen) and NCT 03518697 (clinical trials).

## Introduction

Cystic fibrosis (CF) is an autosomal-recessive disease caused by mutations in the CF transmembrane conductance regulator (CFTR) gene. With an incidence of 1:2500, CF is one of the leading life-limiting genetic disorders in the Western hemisphere [1].

Sleep disturbances and poor sleep quality are common in children, adolescents, and adults with CF, especially in those with advanced lung disease [2, 3]. Coughing, snoring, PEG-feeding, CF-related diabetes, and nocturnal oxygen desaturation can clearly influence the sleeping behavior of CF patients [4]. Participation in regular physical activity plays a crucial role in CF treatment. Regular physical activity, regardless of lung function and age, has a positive effect on the course of the disease [5]. Active patients are characterized by a decreased mortality [6], slower decline in lung function (FEV1) [7], better health-related quality of life (HRQOL) [8], and a better sleep quality [9].

Actigraphy is widely used for long-term estimation of sleep outcomes and daily physical activity at home-based settings [10, 11]. Various studies have demonstrated diagnostic accuracy of actigraphy for measuring sleep parameters using selected scoring settings regardless of the underlying disease, including CF [2, 11–15].

In this study, we analyzed long-term actigraphy-measured sleep and activity data of overall 109 children, adolescents, and adults with CF in a home-based setting. The aim of this analysis was (1) to create a sleep and activity profile and (2) to investigate the relationship between physical activity and sleep quality regarding disease severity and age in a large CF cohort.

## Methods

### Patients

This is a sub-analysis of the CFmobil project, a 12-month partially supervised exercise program for CF patients ≥ 6 years of age, which was conducted from April 2014 to August 2018 at three regional CF centers (University Children’s Hospital Essen, University Children’s Hospital Bochum, and the Adult Cystic Fibrosis Center at the Ruhrlandklinik Essen) in Germany. Inclusion criteria were the diagnosis of CF by (a) two pathological sweat tests (> 60 mmol/L sweat chloride) and/or (b) detection of two disease-defining mutations. Further relevant clinical data was taken from paper or electronically based patient record. We excluded patients with known Cor pulmonale, musculoskeletal diseases, that do not allow continuous exercise training, and patients with an untreated or poorly adjusted CF-related diabetes mellitus. Additionally we excluded patients with incomplete data sets to guarantee a good data quality. All lung function measurements and calculation of BMI were performed in a clinically stable situation at the end of the 4-week actigraphy phase. Forced vital capacity (FVC) and forced expiratory volume in 1 s (FEV1) were measured with a JAEGER MasterScreen Body (CareFusion, Hoechberg, Germany) at all three centers according to ATS guidelines [16]. The study was approved by the ethics committees of the University Duisburg-Essen (14–6117-BO) and Ruhr University Bochum and the study was listed in clinical trials (NCT 03518697). All patients, or their guardians, provided a written informed consent or assent if below 18 years of age.

### Actigraphy

For nondisruptive sleep recording actigraph devices are a powerful device for objective measurement of sleep and activity. In the presented study, we used the wActiSleep-BT Monitor (Actigraph Corp., Pensacola, FL, United States), equipped with a tri-axial acceleration sensor and an ambient light sensor to detect the following sleep parameters: SE, sleep efficiency (%); TIB, total time in bed (min); TST, total sleep time (min); WASO, wake after sleep onset (min); and number of awakenings (*n*). In addition, the following activity-related data was recorded: steps per day, intensity expressed as metabolic equivalents (METs), and subdivided into light (< 3 METs/min/day), moderate (3–5.9 METs/min/day), vigorous (6–8.9 METs/min/day), very vigorous (≥ 9 METs/min/day), and sedentary time (< 1.5 METs/min/day) [17]. All recorded data were averaged over the entire wearing period.

Patients received the device at the beginning of the CFmobil project and were asked to wear the accelerometer at home for a total period of 4 weeks. All patients wore the device on the wrist of the non-dominant hand. Afterwards, it was returned by mail for computer-based evaluation at the study center. Evaluation of recorded raw data was performed by ActiLife software, Version 6.11.9 (Actigraph Corp., Pensacola, FL, United States).

### Statistics

Methods of descriptive statistics (mean ± standard deviation (SD), range) were used for analysis. Correlation was performed with Spearman’s rank test. In order to test differences between means, the Student’s *t* test was used in case of normal distribution; otherwise, the Kruskal-Wallis test was adopted. To examine whether sleep parameters are affected by physical activity, a stepwise multiple linear regression analysis was implemented. Statistical analysis was performed using version 25 of the SPSS statistics package (SPSS Inc., Chicago, USA). A *p* value ≤ 0.05 was considered to be statistically significant.

## Results

Data from a total of 109 CF patients (45 (41%) females: age 22.7 ± 12.0 years) were available for evaluation. Demographics and clinical characteristics of all patients are presented in Table 1. For better discrimination, we formed three subgroups according to age (years) as follows: group 1, ≤ 18; group 2, 19 to 30; and group 3, >30 years and FEV1 (%pred.); group 1, < 40; group 2, 40 to 69; and group, 3 ≥ 70. The different FEV1 groups represent different age groups according to the nature of the disease as follows: mean age in group 1, 31.8 ± 13.2 years; group 2, 24.6 ± 10.5 years; and group 3, 15.5 ± 8.2 years. The average wearing time of the accelerometer was 24 ± 6 days. A total of 2621 days and a total of 3052 nights of recorded data were available for evaluation.

**Table 1:** Demographics and clinical characteristics

	All Subjects (*n* = 109)	Age ≤ 18 (*n* = 42)	Age 19–30 (*n* = 46)	Age > 30 (*n* = 21)
Age, years	22.7 ± 12.0 (6–69)	12.1 ± 3.4 (6–18)	23.6 ± 3.6 (19–30)	41.9 ± 10.5 (31–69)
Gender				
Female, *n* (%)	45 (41)	19 (45)	20 (43)	6 (29)
Male, *n* (%)	64 (59)	23 (55)	26 (57)	15 (71)
Genotype				
F508del homozygous, *n* (%)	56 (51)	21 (50)	27 (59)	8 (38)
F508del heterozygous, *n* (%)	33 (30)	15 (36)	14 (30)	4 (19)
Other, *n* (%)	20 (19)	6 (14)	5 (11)	9 (43)
BMI, kg/m^2^	19.4 ± 3.4 (14–33)	17.7 ± 2.3 (14–22)	19.9 ± 3.0 (15–33)	21.9 ± 3.9 (17–30)
FEV_1_, L	1.9 ± 0.9 (0.6–5.5)	2.0 ± 0.9 (0.6–5.5)	2.0 ± 0.9 (0.6–4.2)	1.5 ± 0.6 (0.6–3.0)
FEV_1_, %pred.	63.0 ± 26.7 (15–126)	85.4 ± 22.1 (39–126)	51.3 ± 18.9 (20–92)	43.4 ± 16.5 (15–78)
FVC, L	2.7 ± 1.1 (0.9–6.5)	2.5 ± 1.1 (0.9–6.5)	3.0 ± 1.2 (1.0–5.5)	2.6 ± 1.0 (0.9–4.6)
FVC, %pred.	75.0 ± 21.9 (20–124)	87.9 ± 16.9 (41–124)	67.9 ± 21.1 (26–102)	61.2 ± 18.1 (20–85)
Pancreatic insufficiency, *n* (%)	95 (87)	39 (93)	40 (87)	16 (76)
*P. aeruginosa* positive, *n* (%)	51 (47)	9 (21)	28 (61)	14 (67)
Cystic fibrosis-related diabetes, *n* (%)	26 (24)	5 (12)	15 (33)	6 (29)
O2-Supplementation, *n* (%)	7 (6)	0 (0)	4 (9)	3 (14)

Results are presented as mean ± standard deviation (SD) and range or number of patients *n* (%). *BMI* body mass index, *FEV1* forced expiratory volume in 1 s, *FVC* forced vital capacity

Table 2 provides an overview of sleep data for all age and FEV1 groups collected by actigraphy. SE was excellent in the examined collective (91±3%). The median TIB was 508±72 min with 68 patients (62 %) showing a TIB above 8 hours. TST across all groups was 464±73 min. The median wake after sleep onset (WASO) was 42±14 min, while the average number of awakenings was 12±5.

**Table 2:** Actigraphy data for all patients and different age and FEV1 groups

			Age (years)		*p* value	FEV1 (%pred.)			*p* value
	All	≤ 18	19–30	> 30		FEV1 < 40	FEV1 40–69	FEV1 ≥ 70	
	(*n* = 109)	(*n* = 42)	(*n* = 46)	(*n* = 21)		(*n* = 24)	(*n* = 43)	(*n* = 42)	
Steps/day	9459 ± 3261	11,061 ± 2388	7986 ± 3113^**^	9479 ± 3684	≤ 0.01	6898 ± 3039	9876 ± 3144^**^	10,496 ± 2756 ^†##^	≤ 0.01
	(1514–17,478)	(6520–16,629)	(1514–14,103)	(3069–17,478)		(1514–12,826)	(3608–17,478)	(4489–16,629)	
Sed	816 ± 217	706 ± 212	889 ± 189^**^	892 ± 191 ^#^	≤ 0.01	976 ± 178	832 ± 195	719 ± 209 ^†##^	≤ 0.01
	(256–1311)	(256–1131)	(502–1311)	(569–1190)		(709–1311)	(256–1221)	(288–1169)	
Light	499 ± 352	492 ± 279	507 ± 415	493 ± 348	ns	552 ± 453	462 ± 321	506 ± 320 ^##^	ns
	(119–1488)	(193–1331)	(119–1488)	(219–1311)		(119–1426)	(179–1488)	(190–1331)	
Moderate	110 ± 86	145 ± 91	82 ± 73^**^	104 ± 78	≤ 0.01	77 ± 80	102 ± 76	137 ± 92	≤ 0.01
	(7–389)	(39–367)	(7–389)	(24–337)		(7–389)	(21–367)	(21–363)	
Vigorous	13 ± 14	13 ± 13	12 ± 14	13 ± 16	ns	8 ± 12	15 ± 16	13 ± 13	ns
	(0–62)	(0–40)	(0–62)	(0–52)		(0–52)	(0–62)	(0–42)	
Very vigorous	3 ± 6	4 ± 7	3 ± 6	2 ± 3	ns	1 ± 2	3 ± 6	4 ± 7	≤ 0.05
	(0–28)	(0–28)	(0–26)	(0–9)		(0–9)	(0–24)	(0–28)	
SE	91 ± 3	92 ± 3	90 ± 3^**^	90 ± 3 ^#^	≤ 0.01	90 ± 3	91 ± 4	92 ± 3 ^#^	≤ 0.05
	(82–98)	(82–98)	(82–97)	(83–96)		(82–96)	(82–97)	(87–98)	
TIB	508 ± 72	545 ± 71	491 ± 61^**^	471 ± 68 ^##^	≤ 0.01	493 ± 77	494 ± 67	531 ± 71	≤ 0.05
	(346–807)	(401–807)	(377–679)	(346–568)		(376–679)	(346–659)	(427–807)	
TST	464 ± 73	504 ± 72	446 ± 59 ^**^	426 ± 68 ^†##^	≤ 0.01	446 ± 74	449 ± 66	490 ± 72 ^†#^	≤ 0.05
	(315–779)	(368–779)	(354–624)	(315–521)		(331–624)	(315–614)	(385–779)	
WASO	42 ± 14	39 ± 15	45 ± 14	44 ± 14	ns	47 ± 15	43 ± 15	39 ± 13	ns
	(15–90)	(15–90)	(16–77)	(18–72)		(18–77)	(16–90)	(15–67)	
# of awakenings	12 ± 5	12 ± 5	11 ± 4	11 ± 5	ns	12 ± 5	11 ± 5	12 ± 5	ns
	(3–24)	(6–24)	(5–24)	(3–21)		(7–24)	(3–24)	(6–24)	

Results are presented as mean ± standard deviation (SD) and range; *FEV1* forced expiratory volume in 1 s (%pred.), *Sed* sedentary time < 1.5 MET/min/day, *light* ≤ 3 MET/min/day, *moderate* 3 to 5.9 MET/min/day, *vigorous* 6–8.9 MET/min/day, *very vigorous* ≥ 9 MET/min/day; *SE* sleep efficiency (%), *TIB* total time in bed (min), *TST* total sleep time (min), *WASO* wake after sleep onset (min), *#* number

*p* value – Kruskal-Wallis test and post-hoc analysis for differences between groups; age: ≤ 18 vs. 19–30 yrs.: *p ≤ 0.05, **p ≤ 0.01; 19–30 yrs. vs. > 30 yrs.: † = ≤0.05, †† = *p* ≤ 0.01; ≤ 18 vs. > 30 yrs. # = *p* ≤ 0.05, ## = *p* ≤ 0.01. FEV1: <40 vs. 40–69: * *p* ≤ 0.05, ** *p* ≤ 0.01; 40–69 vs. ≥ 70 † = *p* ≤ 0.05, †† = *p* ≤ 0.01; < 40 vs. ≥ 70 # = *p* ≤ 0.05, ## = *p* ≤ 0.01. *ns* not significant

CF patients under the age of 18 show the best sleep efficiency (92±3%), the longest TIB (545±71 min) and TST (504±72 min) and the lowest WASO (39±15 min). With increasing age there is a decrease in SE (p≤0.01), TST (p≤0.01) and TIB (p≤0.01) and an increase in WASO (p>0.05). In terms of lung function, CF patients with FEV1 values > 70%pred. have the best SE (92±3%), the highest TST (490±72 min) and TIB (531±71 min) and the lowest WASO (39±13 min). As FEV1 deteriorates, a change in sleeping behavior can be detected. SE (p≤0.05), TST (p≤0.05) and TIB (p>0.05) decrease, whereas WASO (p>0.05) increases. Interestingly, there is no difference in the number of awakenings between the different age and FEV1 groups (all p>0.05)

Table 2 also gives an overview of the surveyed activity parameters for all patients and age and FEV1 subgroups. On average, the participants performed 9459±3261 steps per day. Most of the time was spent in light activity (499±352 min) or in sedentary time (816±217 min). With increasing age daily physical activity decreases (p≤0.01) and sedentary time increases (p≤0.01). Better FEV1 is associated with higher HPA (p≤0.01) and lower sedentary time (p≤0.01). In terms of intensity of physical activity, the age groups hardly differ. Moderate physical activity is lowest in age group 19 – 30 years compared to children and adolescents ≤ 18 and adults > 30 years (p≤0.01). For light, vigorous and very vigorous intensities there are no differences between age groups (all p>0.05). A similar result is found for the different FEV1 groups. Statistically significant differences are found for moderate and very vigorous intensities related to FEV1 (all p≤0.05). No differences were found for light and vigorous intensities (all p>0.05)

Table 3 shows the correlation between age, FEV1, BMI and measured sleep and activity data. Sleep is influenced by age, FEV1 and BMI. Younger age and better FEV1 lead to a better SE and longer TIB and TST (all p≤0.01), whereas a lower BMI is associated with better SE and longer TIB and TST (all p≤0.01). Interestingly neither age, FEV1 nor BMI have an influence on markers of sleep fragmentation (WASO; p>0.05; number of awakenings; p>0.05). Just as with sleep, influences of age, FEV1 and BMI can be found on daily activity (see table 3). Age and FEV1 have the greatest influence on daily activity in its various intensities, whereas BMI seems to play only a minor role here.

**Table 3:** Spearman’s rank correlation coefficient for age, FEV1, BMI, sleep, and activity data

	Age (years)	FEV1 (%pred.)	BMI (kg/m^2^)
SE	− 0.304^**^	0.286^**^	− 0.245^*^
TIB	− 0.440^**^	0.288^**^	− 0.314^**^
TST	− 0.463^**^	0.331^**^	− 0.353^**^
WASO	0.096	0.051	0.089
# of awakenings	− 0.095	0.042	0.021
Steps/day	− 0.263^**^	0.462^**^	− 0.121
Sed	0.372^**^	− 0.444^**^	0.119
Light	− 0.113	0.127	0.052
Moderate	− 0.299^**^	0.381^**^	− 0.110
Vigorous	− 0.035	0.103	0.008
Very vigorous	− 0.080	0.202^*^	0.054

*FEV1* forced expiratory volume in 1 s (%pred.), *BMI* body mass index, *SE* sleep efficiency (%), *TIB* total time in bed (min), *TST* total sleep time (min), *WASO* wake after sleep onset (min), *#* number, *Sed* sedentary time < 1.5 METs/min/day, *light* < 3 MET/min/day, *moderate* 3 to 5.9 MET/min/day, *vigorous* 6–8.9 MET/min/day, *very vigorous* ≥ 9 MET/min/day. *p ≤ 0.05, **p ≤ 0.01

Sleep compared with activity data (see Table 4) show inconsistent results. HPA has little to no effect on sleeping behavior. There is no significant influence of HPA on SE, TIB, TST, WASO, and the number of awakenings (all *p* > 0.05). However, the time spent in different activity intensities shows a strong influence on sleep. SE shows a strong positive correlation to vigorous (*p* ≤ 0.01) and very vigorous (*p* ≤ 0.01) intensities. Light to moderate intensity has no effect on SE (all *p* > 0.05). Markers of sleep fragmentation (WASO, # of awakenings) are inversely correlated to intense activity (all *p* ≤ 0.01) whereas time spent in lower intensities lead to an increase in sleep interruptions (all *p* ≤ 0.01).

**Table 4:** Spearman’s rank correlation coefficient for sleep and activity parameters

	Steps/day	Sed	Light	Moderate	Vigorous	Very vigorous
SE	0.182	− 0.117	0.039	− 0.042	0.315^**^	0.422^**^
TIB	− 0.034	− 0.174	− 0.268^**^	− 0.050	0.057	− 0.014
TST	0.011	− 0.179	− 0.241^*^	− 0.064	0.137	0.095
WASO	− 0.165	0.011	− 0.114	0.072	− 0.328^**^	− 0.459^**^
# of awakenings	0.124	− 0.272^**^	0.381^**^	0.497^**^	− 0.496^**^	− 0.461^**^

*Sed* sedentary time < 1.5 METs/min/day, *light* < 3 MET/min/day, *moderate* 3 to 5.9 MET/min/day, *vigorous* 6–8.9 MET/min/day, *very vigorous* ≥ 9 MET/min/day, *SE* sleep efficiency (%), *TIB* total time in bed (min), *TST* total sleep time (min), *WASO* wake after sleep onset (min), *#* number. *p ≤ 0.05, **p ≤ 0.01

## Discussion

The aim of our work was to investigate the influence of daily PA on sleep of CF patients. To our knowledge, this is the first study to investigate the relationship between sleep quality and habitual physical activity (HPA) in a large mixed-aged CF population using actigraphy over a 4-week period.

Previous studies showed a large variance in the determination of sleep parameters, depending on the size of the examined patient population, age, severity of the disease, measuring period, and the measurement method used [2, 18–20]. SE was excellent (> 90%) in our study across all age and FEV1 groups. Results similar to ours were found by Jankelowitz et al. [14]. They also used accelerometers to investigate sleep in the home environment over a longer period of time (two consecutive weeks) and found normal SE similar to our results. Other groups found worse SE in CF patients which might be caused by the fact that measurements were shorter (only overnight) and therefore give a less realistic picture of sleeping behavior compared with the presented long-term measurements in the home environment of this study [21, 22].

The presented data show that PA influences sleep of CF patients. However, the general PA (steps/day) plays only a minor role. The time spent in higher PA intensities has much more influence on sleep behavior. However, PA with higher intensity is not influenced by age, only to a limited extent by FEV1 and not at all by the total number of steps per day. This finding is supported by the work of Cox et al. [9] who also investigated PA and sleep parameters using accelerometry in adult CF patients. They found that more fragmented sleep was associated with poorer exercise capacity, poorer FEV1, and less physical activity. Moderate to vigorous intensities of activity had a positive effect on sleep quality. Therapeutic interventions aiming to increase PA should not only focus on increasing the number of steps/day but should also specifically promote PA at higher intensities [23].

Similar results have also been described for other lung diseases. Spina et al. [24] analyzed the association between actigraphically assessed sleep measures and next day physical activity in 932 COPD patients. They found that sleep fragmentation was greater in patients with heavy airflow limitations and increased exertional dyspnea. Patients with a better sleep quality, which was defined by a low number of nocturnal awakenings, a TST over 225 min, a sleep efficiency over 91%, and a sleep onset below 57 min spent significantly more time in light (*p* ≤ 0.01) and moderate physical activity (*p* ≤ 0.01). This is in line with our work in CF patients.

The fact that PA and sleep affect HRQOL of CF patients is of importance for clinical practice [8, 25, 26]. Both PA and sleep seem to influence each other. Regularly performed PA may have a positive effect on sleep and may reduce the consequences of poor sleep quality such as daytime sleepiness and fatigue, which in turn leads to increased activity in everyday life. Thus the clinician should encourage the patient to PA, regardless of age and lung function, with the goal to identify suitable activities and not only to promote general daily PA but also to specifically promote activity at higher intensities [27]. Moola et al. could show in their study that PA counseling in children aged 8 to 18 years indeed leads to higher activity levels, reduced sedentary time, and improved HRQOL [28]. Savi et al. [29] demonstrated a positive effect of PA on aerobic fitness in adult CF patients, which is of prognostic value for the course of the disease [30]. Optimization of sleep with the help of improved PA and the resulting improved HRQOL can have a positive effect on the individual fitness level and thus influences the course of the disease in a positive way.

Especially, with regard to possible complications of CF, increased physical activity must be offered under individual counseling based on medical history, personal preferences, and current physical complaints [31].

As in other studies, we found a significant correlation between age, FEV1, and sleep quality [19, 32, 33]. Young patients on average have a better lung function and less comorbidities that associate with sleep disturbances. One of the main reasons for disturbed sleep in CF is nocturnal hypoxia. The occurrence of nocturnal hypoxia is directly associated with FEV1 [34, 35]. Therefore, sleep behavior may also be improved by optimization of CF therapy and its comorbidities. In addition to the progression of the disease with increasing age and the associated decrease in FEV1 negatively affecting PA, altered life circumstances may also play a role for reduced PA. In adult CF patients, work and transport-related activities are obvious reasons for reduced PA in everyday life compared with healthier and younger people [36]. Furthermore, with progress of the lung disease by age a higher need for intravenous therapies during pulmonary exacerbations is associated with reduced PA and increased sedentary time [37]. In our cross-sectional study, we found that a decrease of PA occurs during adolescence and young adulthood—which has been shown before by Britto et al. [38]. This phase of life is crucial in the disease course of CF patients; therefore, CF teams need to be aware of the reduction of PA in this age group to counteract this trend.

Besides the strengths of our study, we need to face some limitations. We did not use questionnaires for subjective assessment of sleep quality. This could provide additional patient-related information besides the objective measurements we performed, to assess the influence of PA on sleep. Worse subjective sleep quality is often reported in CF despite only minor objective changes in sleep architecture [22]. Beyond that, the lack of a control group does not give us the possibility to compare our results with those of healthy people in order to assess how much PA influences sleeping behavior. Day and night hypoxia seems to be a good indicator for sleep quality and the occurrence of sleep disruptions in CF patients. Due to the long measuring period in the home environment, it was not possible for us to collect this data additionally.

## Conclusions

Our results show that physical activity influences sleep of CF patients in a positive way. Stabilization of FEV1 and intensification of physical activity with the aim to spend more time in high intensity physical activity may improve sleep quality of CF patients.

## Abbreviations

#: number
BMI: body mass index
CF: cystic fibrosis
FEV1: forced expiratory volume in 1 s
FVC: forced vital capacity
HPA: habitual physical activity
HRQOL: health-related quality of life
MET: metabolic equivalent
PA: physical activity
PEG: percutaneous endoscopic gastrostomy
PSG: polysomnography
SD: standard deviation
SE: sleep efficiency
TIB: time in bed
TST: total sleep time
WASO: wake after sleep onset
